# Sperm Swimming Velocity Predicts Competitive Fertilization Success in the Green Swordtail *Xiphophorus helleri*

**DOI:** 10.1371/journal.pone.0012146

**Published:** 2010-08-13

**Authors:** Clelia Gasparini, Leigh W. Simmons, Maxine Beveridge, Jonathan P. Evans

**Affiliations:** 1 Dipartimento di Biologia, Università di Padova, Padova, Italy; 2 Centre for Evolutionary Biology, University of Western Australia, Nedlands, Western Australia, Australia; Roehampton University, United Kingdom

## Abstract

Sperm competition is expected to favour the evolution of traits that influence the performance of sperm when they compete to fertilize a female's eggs. While there is considerable evidence that selection favours increases in sperm numbers, much less is known about how sperm quality contributes towards competitive fertilization success. Here, we determine whether variation in sperm quality influences competitive fertilization success in the green swordtail *Xiphophorus helleri*, a highly promiscuous livebearing fish. We use artificial insemination as a method of controlled sperm delivery and show that sperm swimming velocity is the primary determinant of fertilization success when ejaculates from two males compete to fertilize a female's eggs. By contrast, we found no evidence that sperm length had any effect on siring success. We also found no evidence that pre- and postcopulatory sexual traits were phenotypically integrated in this species, suggesting that the previous observation that reproductive skew favours males with high mating rates is unlikely to be due to any direct association between sperm quality and male sexual ornamentation.

## Introduction

Because females typically mate multiply during a single reproductive episode, a male's reproductive fitness will depend not only on his ability to acquire mates, but also the ability of his sperm to compete for fertilizations (sperm competition) [1]. Thus, sexual selection is expected to favour the evolution of traits that maximise an individual's mating rates *and* fertilization success. Consequently, understanding the interplay between pre- and postcopulatory episodes of selection has become an important focus of recent studies. For example, concordant patterns of pre- and postcopulatory sexual selection have been described in species where competitive fertilization success favours highly attractive males [2], [3]. Such patterns may arise when pre- and postcopulatory traits reflect a male's underlying condition [4], or where females mediate the outcome of sperm competition to favour attractive males [5], a mechanism of postcopulatory sexual selection coined ‘cryptic female choice’ [6], [7]. By contrast, other studies have shown that postcopulatory selection acts antagonistically with precopulatory selection, possibly reflecting trade-offs between the males' investment in pre- and postcopulatory traits [8], or evolutionary conflicts between male and female reproductive interests [9]. Indeed, trade-offs may account for negative associations between fertilization success and mating success reported in insects and mammals [10], [11].

In the context of postcopulatory sexual selection, most of the evidence for evolutionary responses to sperm competition centres around traits that influence sperm production rates or ejaculate size (e.g. testis size) [12]. By contrast, there is relatively little direct evidence that selection favours variation in sperm quality, despite comparative studies revealing evolutionary associations between the strength of postcopulatory sexual selection and various traits such as sperm swimming velocity [13], [14], measures of sperm length [15] and the proportion of live sperm per ejaculate (sperm viability) [16]. Yet, it is becoming increasingly apparent that sperm quality plays a greater role in determining fertilization success than previously thought [17]. For example, sperm swimming speed has been shown to positively correlate with sperm competitive fertilizing ability in birds [18], [19] and externally fertilizing fish [20], [21]. Sperm length has also been implicated in mediating competitive fertilization success, although patterns vary among taxa (e.g. shorter sperm confer an advantage in dung beetles) [22]; longer sperm better in salmon [23]) and the limited evidence presented to date makes it impossible to draw general conclusions on the role of sperm length in sperm competition.

In this study we focus on the role of sperm quality in influencing sperm competition and the relationship between male sexual ornamentation and sperm competitiveness in the green swordtail (*Xiphophorus helleri*), a model system in sexual selection [24], [25]. In this livebearing poeciliid fish males have a conspicuous sword-like appendage on their caudal fins, and females exhibit sexual preferences for males with longer swords [24]. Surprisingly, despite intensive research into precopulatory sexual selection in this species [26], sperm competition has never been studied despite recent evidence for high levels of multiple paternity in natural populations [27], [28]. Nevertheless, there is tentative indirect evidence that pre- and postcopulatory sexual selection may work concordantly in this species, which is also consistent with other members of the poeciliid family, and notably the guppy *Poecilia reticulata* [29]. For example, work on natural populations of green swordtails by Tatarenkov et al. [28] reveals that the number of mates per male (estimated as the number of females that produce at least one fry with that male) is correlated with the number of offspring sired (number of offspring sired within a female). One interpretation of this finding is that males that are more successful at obtaining copulations are also those advantaged in sperm competition. Consistent with this idea, Walling et al. [30] reported that female green swordtails are more likely to produce offspring after mating with long-sworded males than with short-sworded males, suggesting a possible fertility advantage by attractive males.

We examined the influence of sperm quality on competitive fertilization success using an artificial insemination protocol specifically devised for green swordtails [31] and implemented in studies of sperm competition in other poeciliid fishes [2], [32]. This method of controlled sperm delivery allows us to control for the potentially confounding effects of relative ejaculate size, mating order and the differential handling of sperm by females due to their perception of male phenotype [33]. We then explored potential phenotypic relationships between the length of the male's caudal ‘sword’ (an important target of precopulatory sexual selection) [24] and sperm traits, and determine whether variation in both male sexual attractiveness and ejaculate traits predict competitive fertilization success in this species.

## Materials and Methods

### Ethics statement

All animal work was conducted according to University of Western Australia's Animal Ethics Committee (Research Integrity Office, permit number 05/100/513).

### Study population and its maintenance

Swordtails were first generation descendents of wild-caught individuals captured from the Irwin River, Western Australia in 2006 [27]. These fish were housed at a constant temperature (26°C) under a light-dark cycle of 9∶15h. Males (*n* = 54) were housed in mixed-sex aquaria (*ca.* 1∶1 sex ratio) until needed, while females were reared in single sex cultures from the onset of sexual maturity to ensure the availability of virgin females for our artificial insemination assays (see below). Fish were fed live cultures of *Artemia* nauplii throughout the experiment.

### Measures of male phenotype

Each male was anaesthetized, photographed for measures of body phenotype (body length and sword length) and manually stripped to obtain sperm samples for sperm swimming velocity assays and artificial insemination (see below). Male phenotypic traits were analysed from digital images of each male (along with a section of ruler for calibration) using UTHSCSA Image Tool (University of Texas Health Science Center, San Antonio, TX, available at http://ddsdx.uthscsa.edu/dig/itdesc.html). We estimated male standard length (the distance from the male's snout to the base of his caudal fin to within 0.01mm) and sword length (distance from the distal margin of caudal peduncle to the distal tip of the sword) from these images.

### Analyses of sperm swimming velocity and sperm length

Sperm samples were collected from anaesthetised males by applying pressure to the abdomen, just anterior to the base of the male's intromittent organ (the gonopodium). As in other poeciliids, sperm are packaged in bundles (spermatozeugmata), which were left intact prior to artificial insemination (see below) and sperm assays.

We used computer-assisted sperm analyses (CASA) to estimate sperm velocity using the CEROS sperm tracker (Hamilton-Thorne Research, Beverly, MA, USA). For each sample, we placed 3–4 spermatozeugmata in a single well of a 12-well multitest slide (MP Biomedicals, Aurora, OH, USA) coated with 1% polyvinyl alcohol (Sigma-Aldrich) to avoid sperm sticking to the glass slide [34]. Sperm velocity was measured on motile sperm (mean ± SE: 165.6±18.0) following activation by 150 mM KCl [35]. This generated a measure of average path velocity (VAP), which estimates the average velocity of sperm cells over a smoothed cell path. Our preliminary analyses of sperm velocity confirmed that all other measures of sperm velocity, including straight line velocity (VSL; the average velocity on a straight line between the start and the end point of the track) and curvilinear velocity (VCL; the actual velocity along the trajectory) were highly correlated with VAP (both *r*-values>0.94, P<0.0001). We therefore restricted our analysis of sperm velocity to VAP, but note that results were unchanged when we used alternative velocity measures in our analysis (results not presented here). The threshold values for defining static cells for VAP measures were predetermined from these preliminary trials at 25 µm/s.

Estimates of total sperm length were taken from digital photographs obtained under a phase-contrast microscope (Leica DM100) at ×400 magnification. A sample of sperm cells, taken from broken down spermatozeugmata and suspended in 5 µl of 0.9% NaCl, was placed within a single well of the multi-well slide and covered with a coverslip. We photographed approximately 10 sperm cells per male (mean: 9.43±0.19 SE; range 5–10) and measured total sperm length (in µm) using Image Tool software.

### Artificial insemination

We artificially inseminated equal numbers of sperm from two randomly paired males (arbitrarily labelled A and B) into an anaesthetized virgin female using an established protocol for poeciliid fishes [2] and originally devised for *X. Helleri* [31]. In each trial a virgin female was anaesthetized and placed in a polystyrene ‘cradle’ with her genital pore exposed. We used a Drummond sequencing pipette (Sigma-Aldrich) to inseminate 40 sperm bundles from each male simultaneously into the female's genital tract. After insemination, each female was revived in conditioned fresh water and placed in 2l plastic tanks until she produced a brood. These tanks contained both live and plastic plants to provide refuges for newborn fish, thereby reducing the chances of filial cannibalism. In total, we performed 32 artificial inseminations, of which 16 resulted in offspring. All of these broods came from females inseminated by unique pairs of putative sires. After parturition, a tissue sample (fin clip) from the female, the two putative fathers and the whole body of the offspring was collected and stored in absolute ethanol for subsequent paternity analyses.

### Paternity analyses

DNA was extracted using a rapid salt-extraction method [36]. The samples were screened using five microsatellite markers, four developed for *Xiphophorus helleri* [37], and one for *X. Montezumae* [38] (see table 1 for details). To allow for the different annealing temperatures, 3 polymerase chain reactions (PCRs) were carried out. The first contained 1× PCR buffer (10mM Tris-HCl pH 8.3, 50mM KCl) (Invitrogen), 1.5mM MgCl2 (Invitrogen), 200µM of each dNTP (Invitrogen), 250nM of Xhe 02 forward primer labelled with 6-Fam (Geneworks) and Xhe 03 forward primer labelled with Ned (Applied Biosystems), the labelled primer diluted 1∶10 with unlabelled primer, 250nM of Xhe 02 and Xhe 03 reverse primers, 0.5units of Platinum Taq polymerase (Invitrogen), and 1–10ng DNA. PCR amplification was performed with cycling conditions as follows: 94°C for 3 minutes, then 35 cycles of 94°C for 35 seconds, 55°C for 35 s and 72°C for 75 s, and finally 72°C for 30 minutes. The second PCR was similar, but with Xhe 15 forward primer labelled with Pet (Applied Biosystems), Xhe 01 forward primer labelled with 6-Fam (Geneworks), the corresponding reverse primers and an annealing temperature of 60°C. The third PCR was also similar, with KonT38 forward primer labelled with PET (Applied Biosystems), KonT38 reverse primer and an annealing temperature of 48°C. The products from each PCR (1.5µl) were combined and analysed on an ABI3730 Sequencer, sized using Genescan-500 LIZ internal size standard and genotyped using Genemapper software (version 3.7). Paternity assignment was made with a maximum likelihood approach using CERVUS 3.0.3 [39], a software program designed for parentage analysis using codominant loci. For parentage analysis, upstream analyses (allele frequency analysis and a simulation of parentage analysis) were carried out first. The results of these analyses were used to determine the suitability of loci for use in parentage analysis and to calculate critical values of likelihood ratios. These were then used with the real data to determine the confidence of parentage assignments. Each family unit was analysed with the female as the known parent and the two males with which she had mated as the candidate parents, all assignments being made at the strict 95% confidence level.

**Table 1:** Characterization of microsatellite loci in *Xiphophorus helleri*.

Locus	*n*	N_A_	bp	H_O_	H_E_	F_(null)_
*KonT38*	187	4	131–170	0.380	0.447	0.080
*Xhe 01*	190	3	192–203	0.468	0.421	−0.048
*Xhe 15*	191	3	219–230	0.471	0.422	−0.049
*Xhe 02*	190	3	269–309	0.563	0.582	0.023
*Xhe 03*	190	5	187–215	0.621	0.659	0.051

The number of individuals that gave an amplification product (*n*) out of 191 individuals genotyped, the number of alleles observed (N_A_), allele size range in base pairs (bp), observed heterozygosity (H_O_), expected heterozygosity (H_E_) and frequency of null alleles (F_Null_) are listed for each locus.

### Statistical analyses

To determine the relationships between male sexual traits (phenotype and sperm) and siring success, we used a generalized linear model with a binomial error distribution and a logit link function. In this analysis, each replicate was weighted by the number of offspring in order to control for uneven brood sizes among replicates. We included the number of offspring sired by the male arbitrarily labelled as ‘B’ as the response variable, and the total number of offspring sired in each brood as the binomial denominator. Due to overdispersion of these data, a dispersion parameter was estimated from the residual deviance in the model [40]. Predictor variables were the difference in trait values between the two putative sires (trait B-trait A) and included differences in sword length, body length, sperm swimming velocity (VAP) and total sperm length. We then employed a backward stepwise deletion of non-significant terms, ensuring that excluded terms did not result in a significant change in deviance in the reduced model. These analyses were performed using GenStat (v.10.2). We then used pairwise correlation analyses to examine the sign and strength of associations among ejaculate and phenotypic traits. We report corrected (Bonferroni) *P*-values associated with these tests as a conservative guide to assessing their significance.

## Results and Discussion

**Figure 1:**
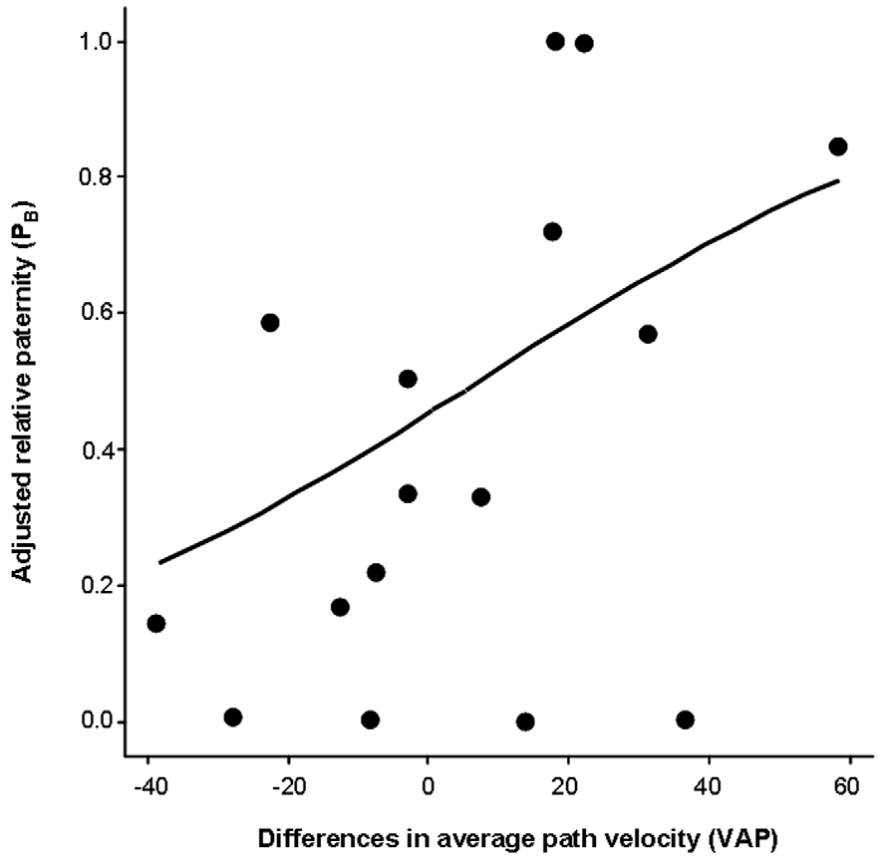
Fitted curve from a generalized linear model analysing the relationship between relative paternity (P_B_: proportion of offspring sired by the male arbitrarily labelled as B in each replicate) and sperm swimming velocity (VAP).

The generalized linear model revealed that variation in VAP was the only significant predictor of paternity success in green swordtails (table 2). None of the other traits (sperm length, male body size and sword length) was included in the final model. We found that males with relatively high VAP scores enjoyed higher paternity success than their rivals who had lower VAP scores (figure 1). This finding therefore adds to a small but growing number of studies revealing similar positive associations between sperm swimming velocity and competitive fertilization success across other taxa (see introduction). Recent unpublished work on the guppy *Poecilia reticulata*, a related poeciliid fish, has similarly uncovered a positive association between sperm swimming velocity and competitive siring success (Boschetto C, Gasparini C, Pilastro A, unpublished manuscript). Consistent with these findings, recent work on natural populations of guppies has revealed that males inhabiting downstream portions of rivers in which polyandry is most prevalent (i.e. where sperm competition is likely to be most intense) have relatively faster swimming sperm than their upstream counterparts, suggesting evolutionary responses to sperm competition at the intraspecific level [41]. To the extent that these findings reflect a more general pattern across livebearing fishes, we predict that macroevolutionary patterns of ejaculate evolution in this group will reveal a positive association between measures of sperm velocity and the strength of selection from sperm competition.

**Table 2:** Proportion of offspring sired by focal male (P_B_) in relation to differences in sperm swimming velocity between rival ejaculates.

Generalized linear model with binomial errors					
	d.f.	Deviance	mean deviance	ratio	*P*
Regression	1	204.4	204.5	9.1	0.009
Residual	14	316.3	22.6		
Total	15	520.8	34.7		

Estimates of parameters				
	estimate	s.e.	*t*_14_	*P*
Constant	−0.19	0.28	−0.7	0.52
Sperm swimming velocity (male B-male A).	0.03	0.009	2.85	**0.013**

We detected no significant effect of sperm length on competitive fertilization success, which contrasts with results on other taxa (see Introduction) and recent comparative studies revealing that measures of sperm length can covary positively with the strength of selection imposed through sperm competition [13], [42]–[44]. We currently lack comparative data revealing macroevolutionary patterns of selection on sperm morphology in livebearing fishes, although recent unpublished data from guppies suggests that the length of the sperm's midpiece is negatively associated with competitive fertilization success (Boschetto C, Gasparini C, Pilastro A, unpublished manuscript).

Interestingly, we detected no significant associations between sword or body length and either of the ejaculate traits (all *r*<0.21, *P*>0.30), although sword length and body length were significantly positively correlated (see table 3 for all pairwise association tests). Thus, based on these analyses we found no evidence that pre- and postcopulatory sexual traits were phenotypically integrated in this species, suggesting that the previous observation that reproductive skew favours males with high mating rates [28] is unlikely to be due to a direct association between sperm quality and either the length of the sword or male body size, both of which are important determinants of male attractiveness in this species [24], [45]. Nevertheless, we cannot rule out the possibility that sperm quality is associated with non-measured sexually-selected traits such as courtship behaviour, which is also a target of pre-copulatory sexual selection in this species [46]. Future work that focuses on behavioural correlates of sperm traits [47] and competitive fertilization success [48] may yet establish such relationships between pre- and postcopulatory sexual selection in green swordtails. Our results also fail to support the recent idea that the higher likelihood of reproduction when females are paired with long sworded males is due to a relationship between sword length and sperm quality [30]. Instead, variation in sperm numbers or insemination success may account for the higher fertility and/or competitive siring success of attractive males. Thus, an interesting avenue for future research would be to determine whether males differ in their ability to transfer sperm to females, and the extent to which any such effect is due to intrinsic differences among males or instead to female-mediated effects, which have previously been reported in guppies [33].

**Table 3:** Pairwise correlation coefficients for pairwise associations (*n* = 54) among the measured traits.

	Body length	Sword length	VAP	Sperm length
Body length	-	**0.718**	−0.191	0.188
Sword length	-	-	−0.189	0.212
VAP	-	-	-	0.077

Significance levels were adjusted using the Bonferroni adjustment; bold values indicate *P*<0.05 following Bonferroni adjustment.

In conclusion, our study reveals that sperm velocity is positively and significantly associated with siring success in sperm competition trials, while sperm length had no measurable influence on paternity success. We also found that measures of male sexual ornamentation did not covary with the measured sperm traits or paternity rates, suggesting that unlike some other poeciliid taxa (notably guppies), male attractiveness, at least based on the measure of sword length used in this study, does not signal sperm quality and cannot account for the higher fertility and siring success of attractive males reported in previous studies.
